# TRAL 2.0: Tandem Repeat Detection With Circular Profile Hidden Markov Models and Evolutionary Aligner

**DOI:** 10.3389/fbinf.2021.691865

**Published:** 2021-06-25

**Authors:** Matteo Delucchi, Paulina Näf, Spencer Bliven, Maria Anisimova

**Affiliations:** ^1^ Institute of Applied Simulations, School of Life Sciences und Facility Management, Zurich University of Applied Sciences, Wädenswil, Switzerland; ^2^ SIB Swiss Institute of Bioinformatics, Lausanne, Switzerland; ^3^ Laboratory for Scientific Computing and Modelling, Paul Scherrer Institute, Villigen PSI, Villigen, Switzerland

**Keywords:** bioinformatics, computational biology, genome annotation, protein sequence analysis, tandem repeats, profile hidden markov models

## Abstract

The Tandem Repeat Annotation Library (TRAL) focuses on analyzing tandem repeat units in genomic sequences. TRAL can integrate and harmonize tandem repeat annotations from a large number of external tools, and provides a statistical model for evaluating and filtering the detected repeats. TRAL version 2.0 includes new features such as a module for identifying repeats from circular profile hidden Markov models, a new repeat alignment method based on the progressive Poisson Indel Process, an improved installation procedure and a docker container. TRAL is an open-source Python 3 library and is available, together with documentation and tutorials *via* vital-it.ch/software/tral.

## 1 Introduction

Recent years have seen an increased awareness of the importance of genomic tandem repeats (TRs) as functional features. TRs are adjacent repetitive units in genomic sequences, they are known for their associations with diseases and immune related functions and often play an important role in nucleic acid binding (Kajava, 2012; Delucchi et al., 2020; Gidley and Parmeggiani, 2021). TRs are found in abundance throughout the three domains of life (Marcotte et al., 1999; Delucchi et al., 2020). Many protein TRs fold in specific structures (Bassot and Elofsson, 2021). A significant fraction of TRs, however, remain unstructured. It is expected that new TRs can originate i. a. by replication slippage (Ellegren, 2004) or by duplication of intrinsically disordered regions (Delucchi et al., 2020).

The evolutionary conservation of many TRs supports their functional importance (Schaper et al., 2014; Delucchi et al., 2020; Chakrabarty and Parekh, 2021). For example, over 60% of mammalian TRs were estimated to be up to 300 Mya old, including well-studied repeats such as armadillo repeat proteins (ArmRP), leucine-rich repeats (LRR), HEAT and PHD-finger. Yet, despite strong conservation of some repeats, TR annotation and analysis remain challenging (Bahlo et al., 2018; Paladin et al., 2021; Chakrabarty and Parekh, 2021), particularly if TR units have diverged over time through indels and point mutations, duplication and loss of TR units, recombination, replication slippage and gene conversion e.g., Tørresen et al. (2019). In contrast, short TRs or microsatellites have been attracting attention as they are a rich source of genetic variability. For example, in the human genome these contribute about 3%, more than the entire protein coding sequences and are highly polymorphic [ >100 times more frequent than point mutations (Willems et al., 2014)]. Short TRs are often used as genetic markers for diagnostics, particularly in cancer research and, it appears, themselves play a role in cancer (Giovannucci et al., 1997; Vega et al., 2001). Longer repeats, however, can also contribute to increased variability. For example, while LRRs are generally conserved in mammals, they were found to display high variety across plant genomes (Schaper and Anisimova, 2015). In plant genomes, LRRs are typically found in resistance genes (R-genes), where gains or losses of TR units potentially contribute to changing resistance properties in response to emerging pathogens.

Tandem Repeat Annotation Library (TRAL) has been developed in order to analyze a variety of in molecular sequences. TRAL implements a variety of tasks for analyzing tandem repeats (Schaper et al., 2015), both in DNA or amino acid sequences. These include: TR annotation using either sequence profiles or *de novo* TR detectors; identification and filtering of overlapping annotations; filtering by statistical significance; and retrieval of TR characteristics such as TR unit length, number, divergence, indel distribution and TR unit alignments (Schaper et al., 2012). TRAL was shown to be highly successful in the analyses of TRs, for example, relating structure and function of TR units to their evolutionary mode (Schaper et al., 2014). Unlike most other predictors, TRAL allows statistical validation of potential TR candidates based on the evolutionary definition of a tandem repeat. By TRAL’s definition, a TR region is statistically significant if its units share common ancestry (evaluated by testing for homology using a likelihood ratio test). For further details of the method we refer the reader to Anisimova et al. (2015), while Schaper et al. (2015) presents an extensive benchmarking of method’s performance in simulations with and without repeats as well as on real data.

Recently we conducted a large-scale survey of protein TRs, which relied on TRAL for TR annotation (Delucchi et al., 2020). This study highlighted the complexity of TR annotations in proteomic sequences and showed the diversity of their functional roles and origins.

## 2 Method

The new release TRAL 2.0 brings new modules to annotate specific TRs across genomes and improved TR filtering by a new scoring function based on the realignment of TR units with an evolutionary aware indel model, the Poission Indel Process (Bouchard-Côté and Jordan, 2013).

### 2.1 Installation Improvements

Following the requests from our users, we provide many usability improvements, including easy installation and packaging. TRAL depends on numerous external software tools (Table 1). In the previous release, all these packages (with heterogeneous support and development status) had to be installed separately. The installation procedure could be time-consuming and difficult. Our new version provides an “easy setup” procedure, which enables installation of all required external software at once or individually on Linux. Additionally, fully configured TRAL environments are available as either a docker container (github.com/acg-team/tral/packages) or a Vagrant box.

**TABLE 1:** External Software utilized by TRAL and installed using easy setup.

Tool	Use	Reference
HHrepID	De novo TR prediction	Biegert and Söding (2008)
Phobos	De novo TR prediction	Mayer (2007)
T-REKS	De novo TR prediction	Jorda et al. (2010)
TRF	De novo TR prediction	Benson (1999)
TRED	De novo TR prediction	Sokol et al. (2007)
TRUST	De novo TR prediction	Szklarczyk and Heringa (2004)
XSTREAM	De novo TR prediction	Newman and Cooper (2007)
Hmmer	HMM construction	Eddy (2009)
ProPIP	Multiple Sequence Alignment	Maiolo et al. (2021)
MAFFT	Multiple Sequence Alignment	Katoh et al. (2002)
PhyML	Phylogenetic inference	Guindon and Gascuel (2003)
ALF	Simulating repeat sequences	Dalquen et al. (2012)

TRAL is written in Python version 3.6, and is available open-source under the GPL-2.0 license together with the much improved comprehensive documentation and tutorials on: github.com/acg-team/tral. External packages are governed by their own license terms and conditions.

We provide tutorials that explain how to interpret the output of TRAL. Examples are provided to illustrate *de novo* and cpHMM based TR annotation, statistical evaluation and filtering of the candidate repeats. Further, we provide explanations of the usage of command line tools to retrieve repeats from sequence databases and deal with large sequence data sets.

### 2.2 Circular Profile HMM Search Module

**FIGURE 1:**
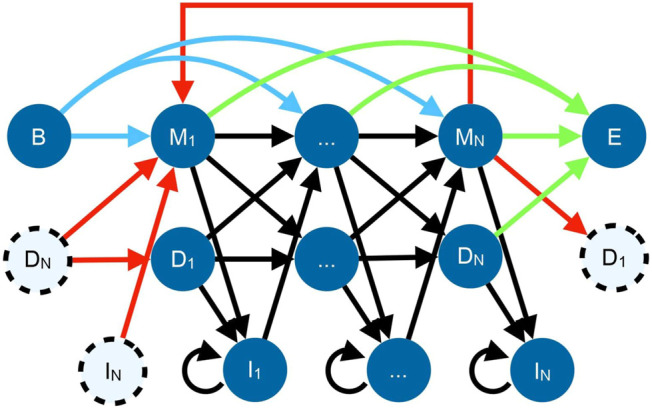
State diagram for the cpHMM (Schaper et al., 2014). Black arrows indicate standard profile HMM transitions within a repeat, with each amino acid corresponding to a match ( $M_{i}$ ), deletion ( $D_{i}$ ), or insertion ( $I_{i}$ ). Red arrows implement the circular permutation by transitioning from the end of the repeat to the beginning (some states are repeated in dashes for simplicity). Since repeats may start and end at any position, equal-weight transitions connect the begin state (*B*, blue arrows) and end state (*E*, green arrows) with every match state.

TRAL has traditionally focused on *de novo* repeat identification, i.e, without the prior knowledge about a potential TR unit (such as the TR unit length and its profile). This is useful for studying repeats in proteins that are expected to have repeated adjacent units but have not been annotated yet. The new search package provides methods for identifying and analyzing a specific repeat (defined by its amino acid or DNA profile) across one or more genomes. TR units in TRAL are represented using a circular profile hidden Markov model (cpHMM) (Figure 1) (Schaper et al., 2014). These can be generated from *de novo* searches or from other sources, such as Pfam alignments of known repeats.

TRAL systematically detects the query cpHMM in a database of sequences, and allows filtering results by repeat length and statistical significance. A log-odds ratio is reported for each result, giving the cpHMM match probability normalized by the probability of a match to a random sequence. Searches of cpHMMs can be run either programmatically or using a command line tool.

**FIGURE 2:**
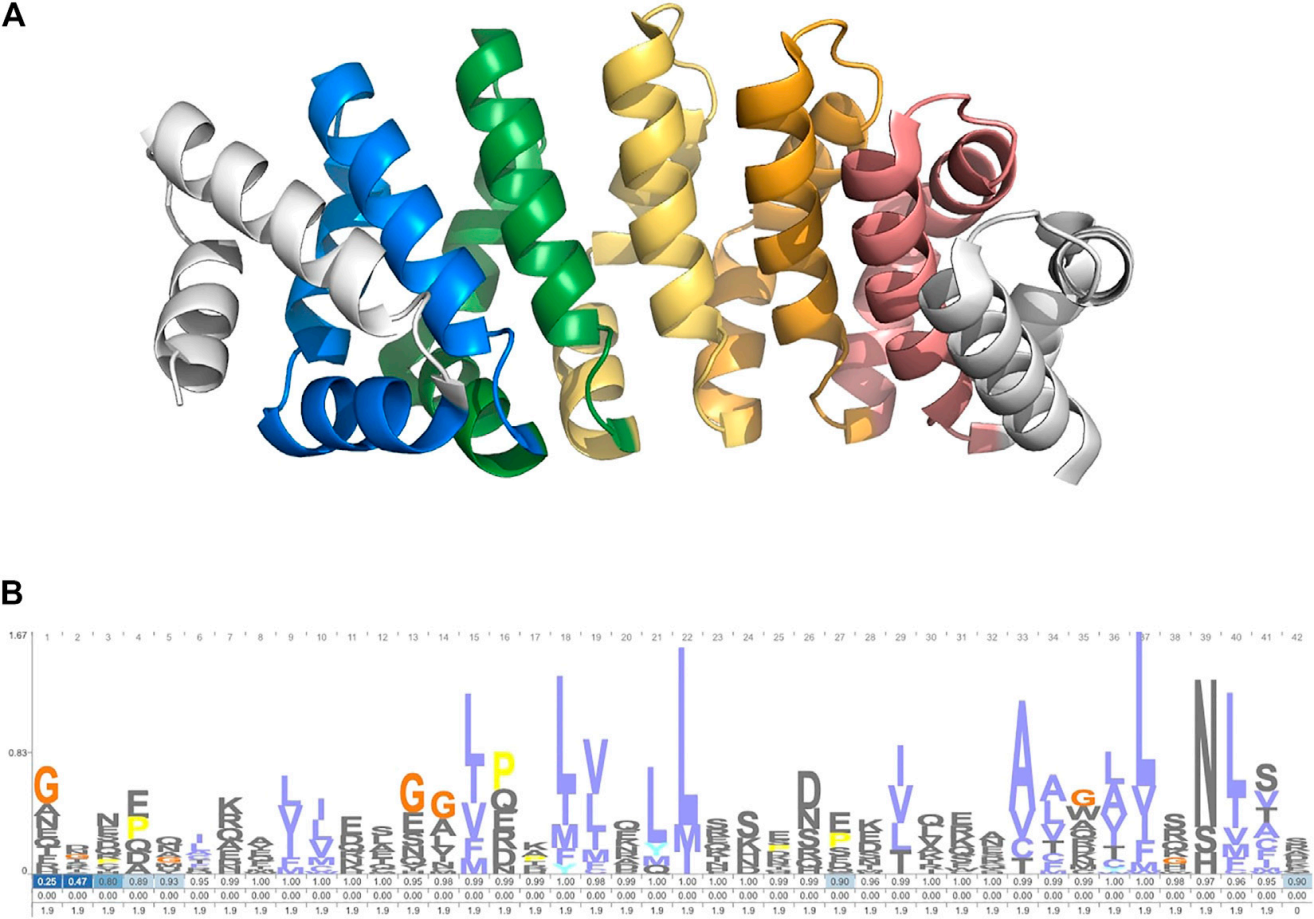
**(A)** Designed ArmRP YIIIM5AII (PDB: 5AEI) (Hansen et al., 2016). **(B)** Logo showing the cpHMM emmission and transmission probablities. Created with Skylign (Wheeler et al., 2014).

The search module was used to identify members of the armadillo repeat protein family. This 42-residue repeat is well characterized and can be found across metazoan lineages. It forms a conserved alpha-barrel structure (Figure 2A). A cpHMM was constructed based on the alignment in Gul et al. (2017) (Figure 2B). TRAL was used to identify armadillo repeats across a selection of 94 metazoan species. Species were taken from the 15% Representative Proteome Group [2018_03 release; https://proteininformationresource.org/rps/; Chen et al. (2011)].

### 2.3 Evolutionary Indel TR Unit Alignment

To filter out spurious annotations, TRAL uses scoring functions based on evolution models. Falsely filtering out a true TR may happen due to either unsuitability of the TR scoring function for TR classification, or inaccurate annotation of the predicted TR units, location, or unit alignment. Thus, better TR unit alignments should contribute to improving the TR prediction quality. Unit alignments are relatively short and are often dominated by short indels, making it difficult to accurately annotate TR unit borders. To improve the annotation of borders, we integrated a new alignment method for realigning TR units with an explicit evolutionary indel model - the progressive PIP (Maiolo et al., 2018) based on the PIP model (Bouchard-Côté and Jordan, 2013).

As modeling of indel evolution is computationally challenging, the dynamics of indels are typically not modeled explicitly, e.g., in the popular aligner MAFFT (Katoh et al., 2002), which was already integrated in TRAL to realign TR units.

The realignment of TR units can be performed after detecting TRs (*de novo* or based on an HMM) or for any given annotated TR. This allows for an iterative refinement of cpHMMs.

The realignment module allows the realignment of any given TR with MAFFT (default) or progressive PIP, ProPIP (Maiolo et al., 2021). When using progressive PIP, a TR unit phylogeny is inferred using PhyML (Guindon et al., 2010) from the initial unit alignment and used as a guide-tree when realigning with progressive PIP. The insertion and deletion rates (λ and μ) are estimated from the initial alignment. By default the indel rate is constant, and a variable indel rate option uses the γ-distribution with the shape parameter α=0.5 . Note that for PIP-based alignment, with fewer than 3 TR units, a star tree is initialized and an initial alignment should have at least one gap.

## 3 Results

### 3.1 Detected Armadillo Repeat Proteins

TRAL was able to identify ArmRP members in all 94 species (Supplementary Figure S1). The number identified per species varied between 14 and 170, with a mean of 51 proteins per species (Supplementary Table S1). In humans, 107 ArmRP members were identified. Distinguishing ArmRP from related families such as HEAT proteins is difficult without structural information, but this result is comparable to other databases: Interpro annotates 127 human ArmRP in the “Armadillo” superfamily (IPR000225), while SMART includes 73 domains (SM00185).

### 3.2 Tandem Repeat Unit Alignment

**FIGURE 3:**
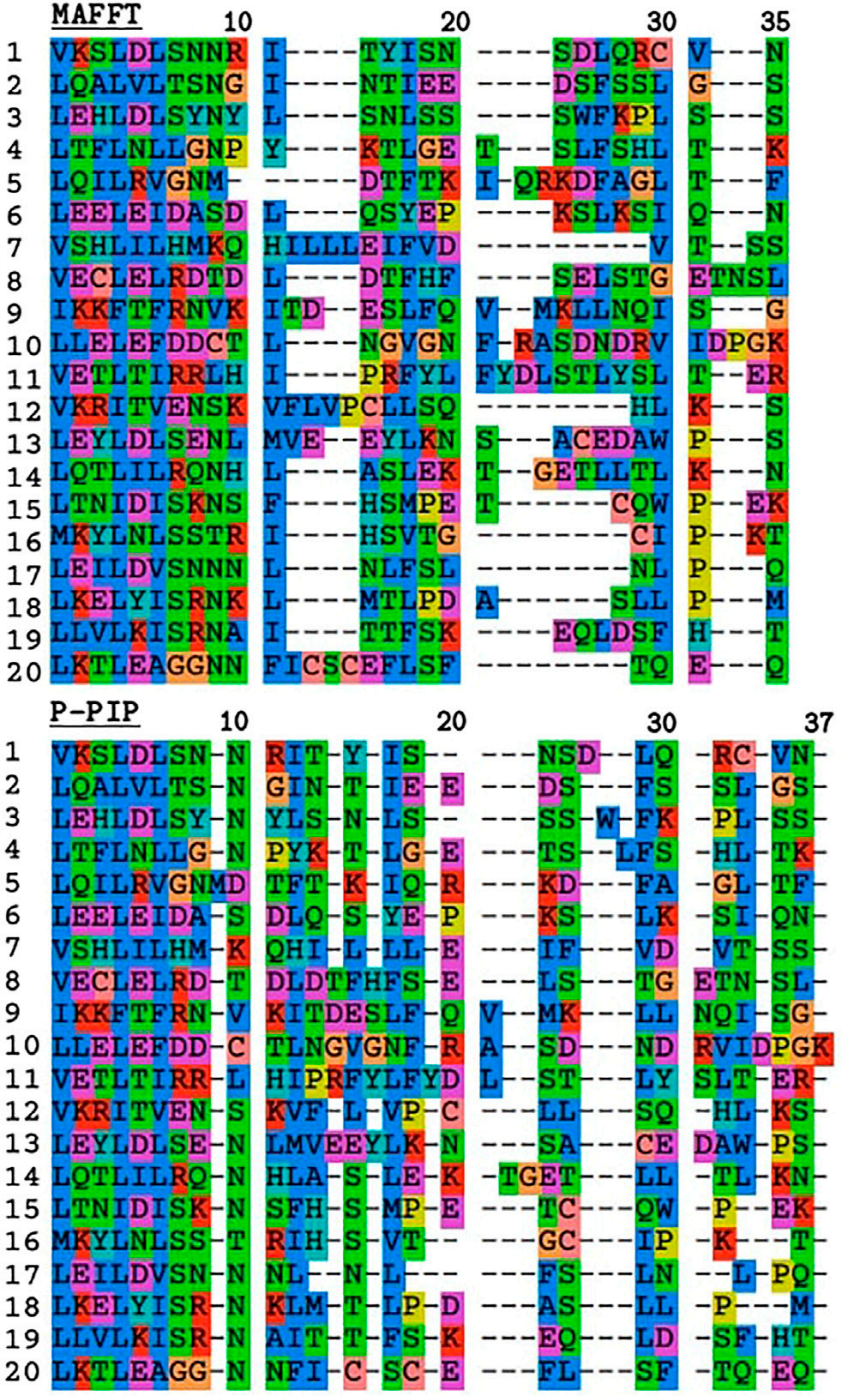
Comparison of TR unit alignments inferred with MAFFT and progressive PIP for leucine-rich repeats from TLR2. The first 11 residues correspond to the highly conserved segment with the motif LxxLxLxxNxL where “L” is Leu, Ile, Val or Phe, and “N” is Asn, Thr, Ser, or Cys (Matsushima et al., 2007).

To demonstrate the effect of the indel model on TRAL results, we used TRAL with default settings to realign the leucin-rich repeat units from the toll-like receptor TLR2 with MAFFT and ProPIP (Figure 3). The PIP-based alignment was slightly longer compared to MAFFT’s, which is consistent with the “phylogeny-aware” nature of this method preventing “overalignment”. The overall divergence was significantly lower, showing higher residue conservation in gap regions compared to MAFFT. This is consistent with (Maiolo et al., 2018), who showed that progressive PIP method infers gap patterns similar to those inferred by PRANK (Löytynoja, 2014), and also phylogenetically meaningful as supported by empirical studies e.g., Abram et al. (2010). While penalizing indels in aligners remains subjective, including the progressive PIP in TRAL allows an alternative method to be studied, particularly in TRs dominated by short indels.

## 4 Discussion

With TRAL version 2.0 the tool became significantly easier to use and gained significant new features. Profile based TR detection is now easy and automated, allowing the investigation of TR defined by a specific query sequence or a provided profile model. The integration of TR unit alignment based on an explicit evolutionary aware indel model should help prevent unit over-alignment, contributing to the increased TR prediction quality. Moreover, the iterated refinement TR annotation allows to consider alternative views of indel evolution affecting the detected TRs.

Some limitations of TRAL remain. TRAL relies heavily on external software for identifying repeats, and many tools struggle with poorly conserved repeats. However, a plugin system exists to allow incorporating future advances in repeat detection into the TRAL framework. The statistical model used in TRAL was primarily used for protein alphabets. While TRAL can also be applied to polynucleotide sequences, the restricted alphabet limits the power such that significance thresholds may need to be adjusted for highly divergent sequences. For protein-coding sequences, in the future, we consider adding a codon alphabet to improve the power of detection. This may also bring additional advantages of being able to analyze protein coding repeats at the level of synonymous DNA changes [e.g., see section 4.3 of Kosiol and Anisimova (2019)].

Augmented with user-friendly documentation, tutorials, the simplified installation and independence of operating systems, TRAL 2.0 addresses a wider range of researchers.

## Data Availability

Publicly available datasets were analyzed in this study. This data can be found here: https://github.com/acg-team/tral.
