# Supplementary material for: TRAL 2.0: Tandem Repeat Detection With Circular Profile Hidden Markov Models and Evolutionary Aligner
Source: Front Bioinform. 2021 Jun 25;1:691865. doi: 10.3389/fbinf.2021.691865 (PMC9581039; doi:10.3389/fbinf.2021.691865)
Supplement: Supplementary file 2 [file Image1.pdf]

Supplementary Material

1 SUPPLEMENTARY DATA

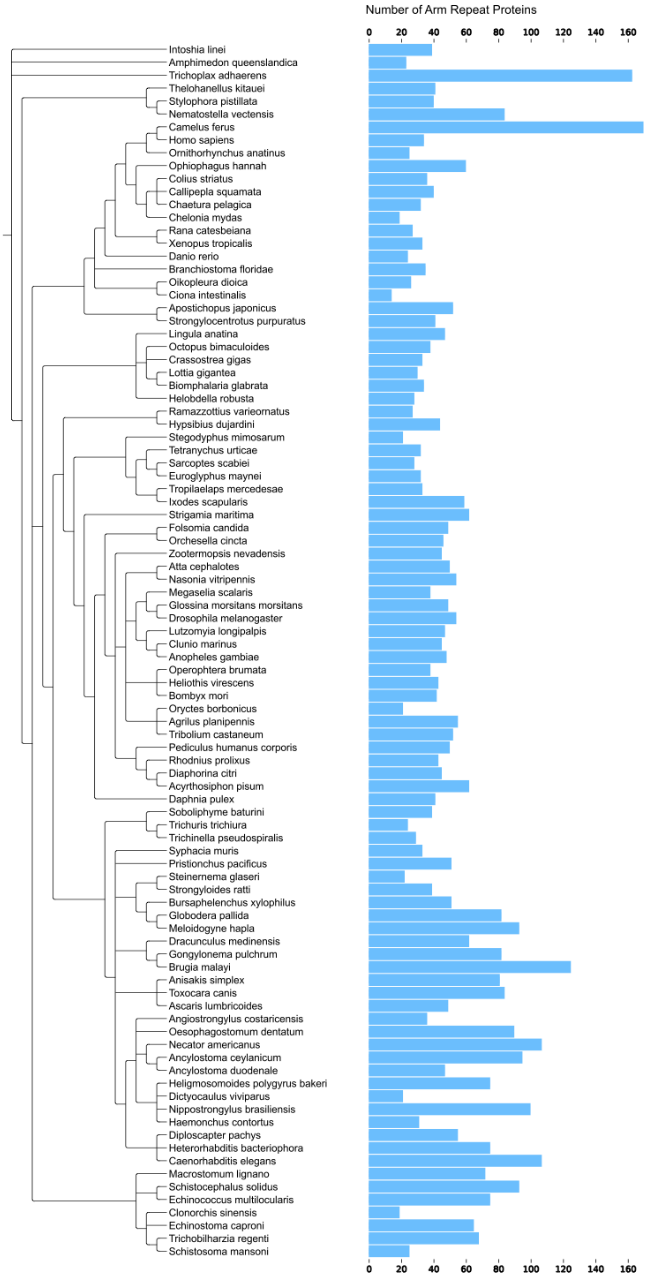

Figure S1. Prevalence of ArmRP members across metazoan species. The phylogenetic tree was generated by the NCBI CommonTree tool (`https://www.ncbi.nlm.nih.gov/Taxonomy/CommonTree/wwwcmt.cgi`).
